# MAP-RSeq: Mayo Analysis Pipeline for RNA sequencing

**DOI:** 10.1186/1471-2105-15-224

**Published:** 2014-06-27

**Authors:** Krishna R Kalari, Asha A Nair, Jaysheel D Bhavsar, Daniel R O’Brien, Jaime I Davila, Matthew A Bockol, Jinfu Nie, Xiaojia Tang, Saurabh Baheti, Jay B Doughty, Sumit Middha, Hugues Sicotte, Aubrey E Thompson, Yan W Asmann, Jean-Pierre A Kocher

**Affiliations:** 1 Department of Health Sciences Research, Mayo Clinic, 200 First Street SW, Rochester, MN 55905, USA; 2 Department of Cancer Biology, Mayo Clinic, 4500 San Pablo Road, Jacksonville, FL 32224, USA; 3 Department of Health Sciences Research, Mayo Clinic, 4500 San Pablo Road, Jacksonville, FL 32224, USA; 4 Present Address: Department of Health Sciences Research, Mayo Clinic, 200 First Street SW, Rochester, MN 55905, USA

**Keywords:** Transcriptomic sequencing, RNA-Seq, Bioinformatics workflow, Gene expression, Exon counts, Fusion transcripts, Expressed single nucleotide variants, RNA-Seq reports

## Abstract

**Background:**

Although the costs of next generation sequencing technology have decreased over the past years, there is still a lack of simple-to-use applications, for a comprehensive analysis of RNA sequencing data. There is no one-stop shop for transcriptomic genomics. We have developed MAP-RSeq, a comprehensive computational workflow that can be used for obtaining genomic features from transcriptomic sequencing data, for any genome.

**Results:**

For optimization of tools and parameters, MAP-RSeq was validated using both simulated and real datasets. MAP-RSeq workflow consists of six major modules such as alignment of reads, quality assessment of reads, gene expression assessment and exon read counting, identification of expressed single nucleotide variants (SNVs), detection of fusion transcripts, summarization of transcriptomics data and final report. This workflow is available for Human transcriptome analysis and can be easily adapted and used for other genomes. Several clinical and research projects at the Mayo Clinic have applied the MAP-RSeq workflow for RNA-Seq studies. The results from MAP-RSeq have thus far enabled clinicians and researchers to understand the transcriptomic landscape of diseases for better diagnosis and treatment of patients.

**Conclusions:**

Our software provides gene counts, exon counts, fusion candidates, expressed single nucleotide variants, mapping statistics, visualizations, and a detailed research data report for RNA-Seq. The workflow can be executed on a standalone virtual machine or on a parallel Sun Grid Engine cluster. The software can be downloaded from http://bioinformaticstools.mayo.edu/research/maprseq/.

## Background

Next generation sequencing (NGS) technology breakthroughs have allowed us to define the transcriptomic landscape for cancers and other diseases [1]. RNA-Sequencing (RNA-Seq) is information-rich; it enables researchers to investigate a variety of genomic features, such as gene expression, characterization of novel transcripts, alternative splice sites, single nucleotide variants (SNVs), fusion transcripts, long non-coding RNAs, small insertions, and small deletions. Multiple alignment software packages are available for read alignment, quality control methods, gene expression and transcript quantification methods for RNA-Seq [2-5]. However, the majority of the RNA-Seq bioinformatics methods are focused only on the analysis of a few genomic features for downstream analysis [6-9]. At present there is no comprehensive RNA-Seq workflow that can simply be installed and used for multiple genomic feature analysis. At the Mayo Clinic, we have developed MAP-RSeq - a comprehensive computational workflow, to align, assess and report multiple genomic features from paired-end RNA-Seq data efficiently with a quick turnaround time. We have tested a variety of tools and methods to accurately estimate genomic features from RNA-Seq data. Best performing publically available bioinformatics tools along with parameter optimization were included in our workflow. As needed we have integrated in-house methods or tools to fill in the gaps. We have thoroughly investigated and compared the available tools and have optimized parameters to make the workflow run seamlessly for both virtual machine and cluster environments. Our software has been tested with paired-end sequencing reads from all Illumina platforms. Thus far, we have processed 1,535 Mayo Clinic samples using the MAP-RSeq workflow. The MAP-RSeq research reports for RNA-Seq data have enabled Mayo Clinic researchers and clinicians to exchange datasets and findings. Standardizing the workflow has allowed us to build a system that enables us to investigate across multiple studies within the Mayo Clinic. MAP-RSeq is a production application that allows researchers with minimal expertise in LINUX or Windows to install, analyze and interpret RNA-Seq data.

## Implementation

MAP-RSeq uses a variety of freely available bioinformatics tools along with in-house developed methods using Perl, Python, R, and Java. MAP-RSeq is available in two versions. The first version is single threaded and runs on a virtual machine (VM). The VM version is straightforward to install. The second version is multi-threaded and is designed to run on a cluster environment.

### Virtual machine

Virtual machine version of MAP-RSeq is available for download at the following URL [10]. This includes a sample dataset, references (limited to chromosome 22), and the complete MAP-RSeq workflow pre-installed. Virtual Box software (free for Windows, Mac, and Linux at [11]) needs to be installed in the host system. The system also needs to meet the following requirements: at least 4GB of physical memory, and at least 10GB of available disk. Although our sample data is only from Human Chromosome 22, this virtual machine can be extended to the entire human reference genome or to other species. However this requires allocating more memory (~16GB) than may be available on a typical desktop system and building the index references files for the species of interest.

Tables  1 and 2 shows the install and run time metrics of MAP-RSeq in virtual machine and Linux environments respectively. For Table  2, we downloaded the breast cancer cell line data from CGHub [12] and randomly chose 4 million reads to run through the QuickStart VM. It took 6 hours for the MAP-RSeq workflow to complete. It did not exceed the 4GB memory limit, but did rely heavily on the swap space provided; making it run slower than if it would have had more physical memory available. Job profiling indicates that the system could have used 11GB of memory for such a sample.

**Table 1:** MAP-RSeq installation and run time for QuickStart virtual machine

**QuickStart VM**	**File size**	**Timeline**
Download	2.2GB	~ 20 minutes to download on consumer grade internet
Unpacked size	8GB	-
Time to import into VM	-	~ 10 minutes
VM boot	-	3 minutes
Run time with sample data (chr22 only)	-	~ 30 minutes

**Table 2:** MAP-RSeq installation and run time in a Linux environment

**Linux**	**File size**	**Timeline**
Download	930 MB	~10 minutes to download on consumer grade internet
Install time	-	~6 hours (mostly downloading and indexing references)
Unpacked size	9GB	-
Run time	-	Depends on the sample data used

### Sun grid engine

MAP-RSeq requires four processing cores with a total of 16GB RAM to get optimal performance. It also requires 8GB of storage space for tools and reference file installation. For MAP-RSeq execution the following packages such as JAVA version 1.6.0_17 or higher, Perl version 5.10.0 or higher, Python version 2.7 or higher, Python-dev, Cython, Numpy and Scipy, gcc and g++ , Zlib, Zlib-devel, ncurses, ncurses-devel, R, libgd2-xpm, and mailx need to be preinstalled and referenced in the environment path. It does also require having additional storage space for analysing input data and writing output files. MAP-RSeq uses bioinformatics tools such as BEDTools [13], UCSC Blat [14], Bowtie [15], Circos [16], FastQC [17], GATK [18], HTSeq [19], Picard Tools [20], RSeqQC [21], Samtools [22], and TopHat [23]. Our user manual and README files provide detailed information of the dependencies, bioinformatics tools and parameters for MAP-RSeq. The application requires configuration, such as run, tool and sample information files, as described in the user manual.

Table  3 shows the processing time of the workflow across different sequencing read depths. Time was recorded from a server with 8 quad core Intel Xeon 2.67 GHz processors and 530 GBs of shared memory using Centos 6. For a sample with 1 million reads, MAP-RSeq completes in less than 2 hours. For samples with 150 million to 300 million reads, MAP-RSeq completes in 12-48 hours depending on the hardware used.

**Table 3:** Wall clock times to run MAP-RSeq at different read counts

**MAP-RSeq processing time**	**Read counts**
118 minutes	1000000
82 minutes	500000
71 minutes	200000

## Results and discussion

**Figure 1:**
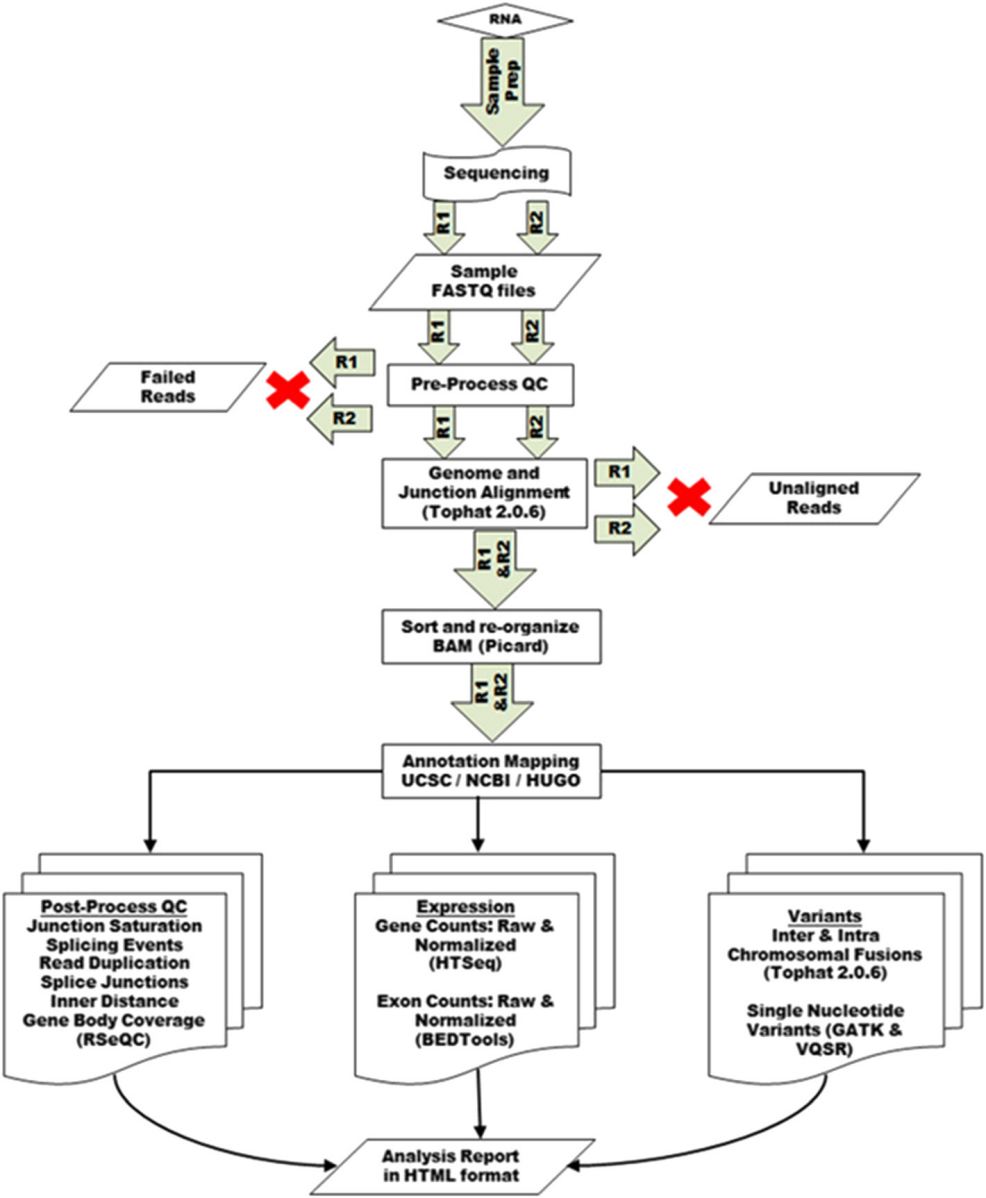
**Flowchart of the MAP-RSeq workflow.** High-level representation of the MAP-RSeq workflow for processing RNA-Seq data.

NGS technology has been outpacing bioinformatics. MAP-RSeq is a comprehensive simple-to-use solution for analysis of RNA-Sequencing data. We have used both simulated and real datasets to optimize parameters of the tools included in the MAP-RSeq workflow. The high-level design of MAP-RSeq is shown in Figure  1. MAP-RSeq consists of the six major modules such as alignment of reads, quality assessment of sequence reads, gene expression and exon expression counts, expressed SNVs from RNA-Seq, fusion transcript detection, summarization of data and final report.

**Figure 2:**
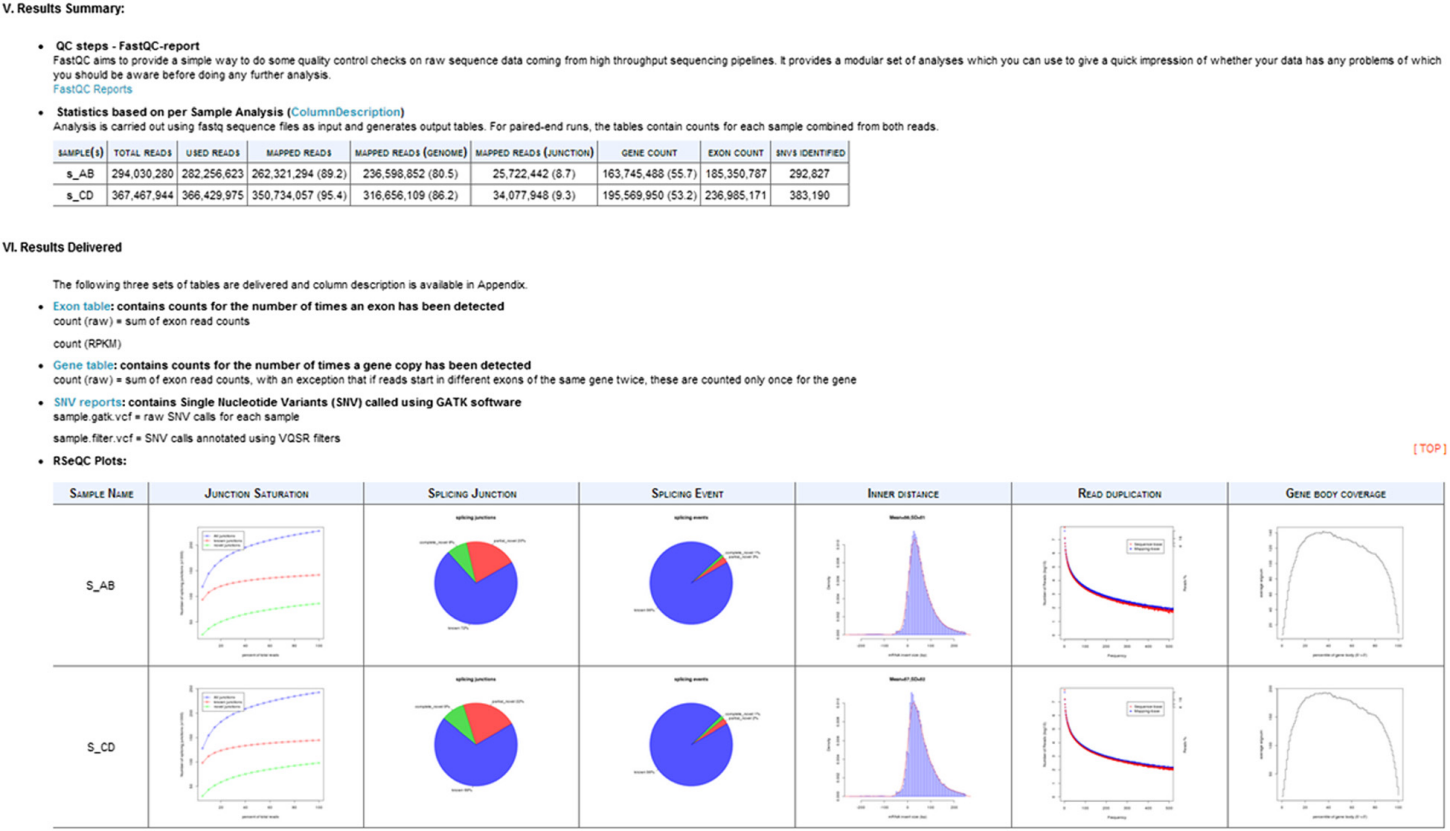
**Screenshot output report (html) of MAP-RSeq.** An example screenshot report of MAP-RSeq output file.

Reads are aligned by TopHat 2.0.6 [23] against the human reference genome build (default = hg19) using the bowtie1 aligner option. Bowtie is a fast memory efficient, short sequence aligner [15]. The remaining unaligned reads from Bowtie are used by TopHat to find splice junctions and fusions. At the end of the alignment step, MAP-RSeq generates binary alignment (BAM) and junction bed files for further processing. The workflow uses the RSeQC software [21] to estimate distance between paired-end reads, evaluate sequencing depth for alternate splicing events, determine rate of duplicate reads, and calculate coverage of reads across genes as shown in the example report file (Figure  2). The summary statistics and plots generated by MAP-RSeq workflow are used for further quality assessments. The example MAP-RSeq result set (files and summary report) from a RNA-Sequencing run can be downloaded from the MAP-RSeq homepage [10].

Several research and clinical projects [24-26] at Mayo Clinic have applied MAP-RSeq workflow for obtaining gene expression, single nucleotide variants and fusion transcripts for a variety of cancer and disease related studies. Currently there are multiple ongoing projects or clinical trial studies for which we generate both RNA-Sequencing and exome sequencing datasets at the Mayo Clinic Sequencing Core. We have developed our RNA-Seq and DNA-Seq workflows such that sequencing data can be directly supplied to the pipelines with less manual intervention. Analysis of the next generation sequencing datasets along with phenotype data enable further understanding of the genomic landscape to better diagnose and treat patients.

### Gene expression and exon expression read counts

A Gene expression count is defined as the sum of reads in exons for the gene whereas an exon expression count is defined as the sum of reads in a particular exon of a gene. Gene expression counts in MAP-RSeq pipeline can be obtained using HTSeq [19] software (default) or featureCounts [27] software. The gene annotation files were obtained from the Cufflinks website [28]. Exon expression counts are obtained using the intersectBed function from the BEDTools Suite [13].

**Figure 3:**
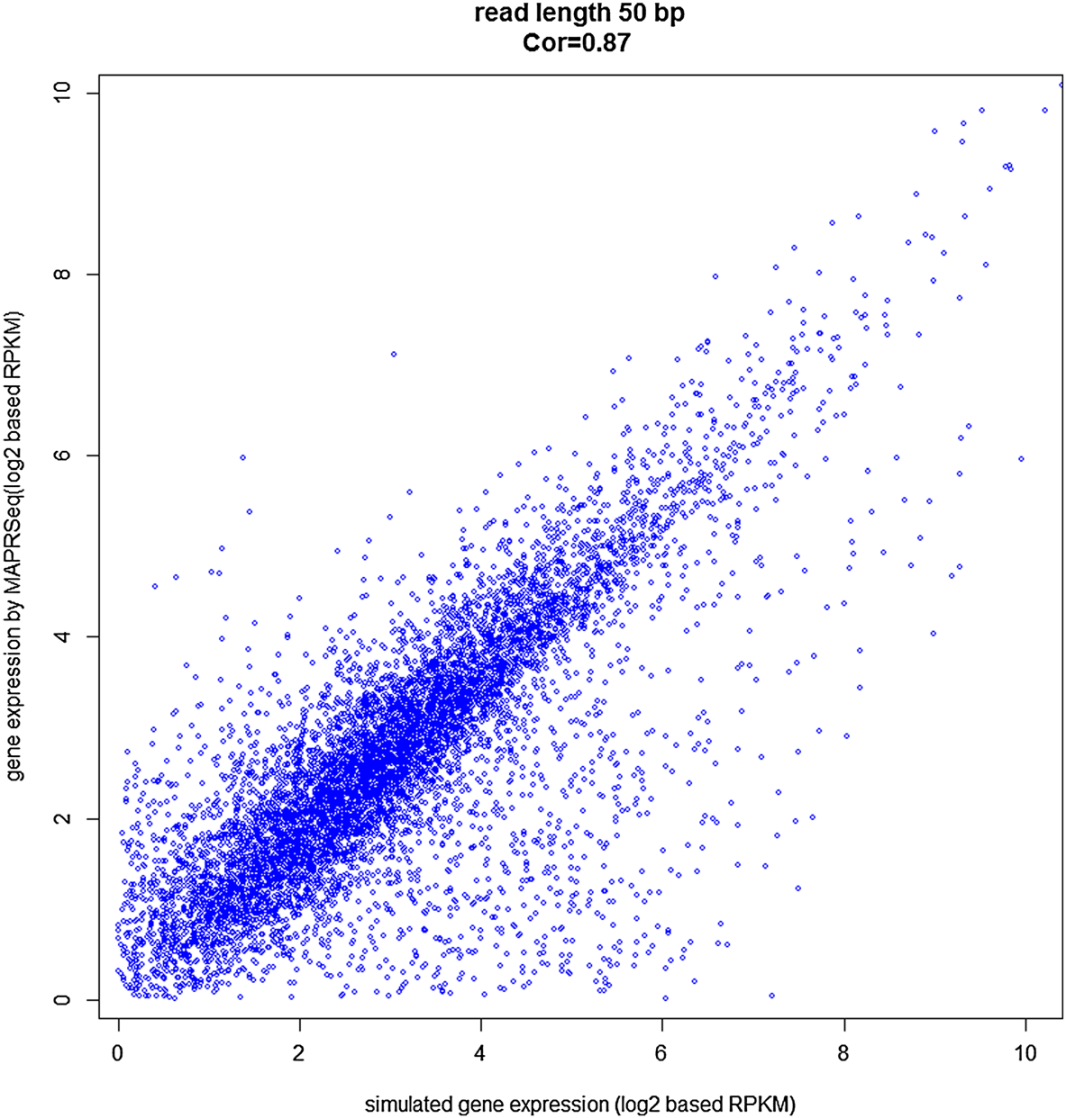
**Correlation of gene counts reported by MAP-RSeq in comparison to counts simulated by BEERS.** MAP-RSeq uses the HTSeq software to classify reads to genomic features. The intersection nonempty mode of HTSeq was applied and the query-name sorted alignment (BAM) file along with the reference GTF file obtained from BEERS were provided as input files to HTSeq for accurate assignment of paired-end reads to genomic features. Comparison of the gene counts (RPKM) obtained from MAP-RSeq with counts for respective genes simulated by BEERS yielded a Pearson correlation of 0.87. The genomic regions where gene expression reported by HTSeq did not completely correlate with simulated expression are due to ambiguous reads or due the fact that either mate of the paired-end read mapped to a different genomic feature, thus categorizing the read as ambiguous by HTSeq.

**Figure 4:**
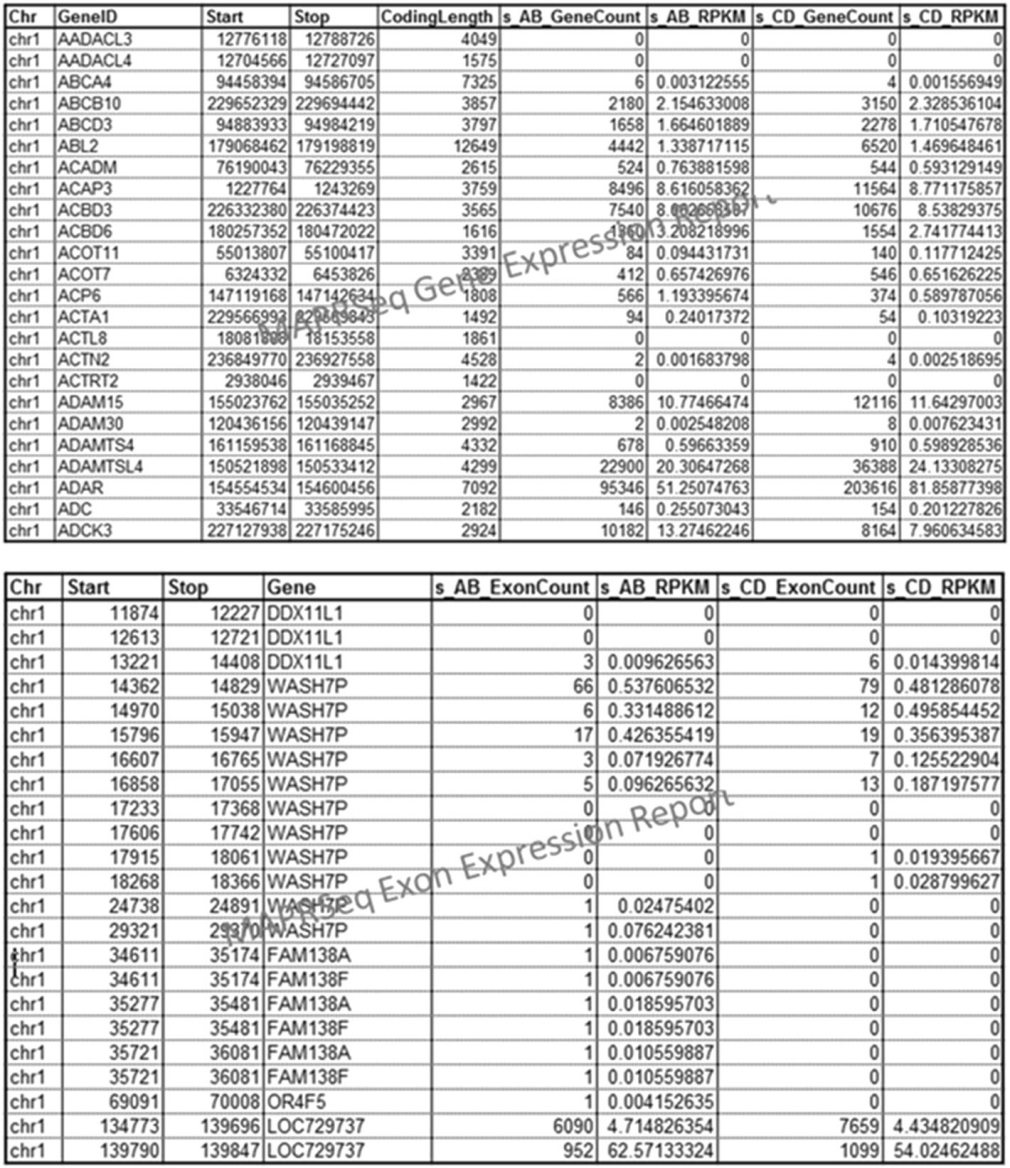
**Screenshots of gene and exon expression reports by MAP-RSeq.** An example of the gene and exon expression counts from the output reports of MAP-RSeq.

MAP-RSeq gene expression counts module was validated using a synthetic dataset for which RNA-Seq reads were simulated using the BEERS software - a computational method that generates paired-end RNA-sequencing reads for Illumina platform [29]. The parameters used for BEERS to generate simulated data are: total reads = 2 million reads, hg19 annotation from RefSeq, read length = 50 bases, base error = 0.005 and substitution rate = 0.0001. Simulated reads were aligned and mapped using the MAP-RSeq workflow. The mapped reads were then input into HTSeq for gene expression counts. Genes with fewer than 30 reads were excluded from the analysis. A correlation of r = 0.87 was observed between the Reads Per Kilobase per Million (RPKM) simulated gene counts and the counts reported by MAP-RSeq, as shown in Figure 3. For simulated data (50 bases), Table  4 summarizes various statistics reported by the MAP-RSeq workflow regarding the alignment of reads to transcriptome and junctions, gene and exon abundance as well as number of SNVs identified and annotated using GATK. An example of MAP-RSeq gene counts table, exon counts table, and normalized counts (RPKM) along with annotations for each run are shown in Figure  4.

**Table 4:** Alignment statistics from MAP-RSeq using simulated dataset from BEERS

**MAP-RSeq features**	**Statistics**
Total number of single reads	4000000
Reads used for alignment	3999995
Total number of reads mapped	3851539 (96.3%)
Reads mapped to transcriptome	3401468 (85.0%)
Reads mapped to junctions	450071 (11.3%)
Reads contributing to gene abundance	1395844
Reads contributing to exon abundance	11266392
Number of SNVs identified	6222

### Differential expression

Each sample is associated to a phenotype, such as tumor, normal, treated, control, etc and that meta-data needs to be obtained to form groups for differential expression analysis. To remove any outlier samples, it is required to perform detailed quality control checks prior to gene expression analysis. There are a variety of software packages that are used for differential expression analysis using RNA-Seq gene expression data [4,30-32]. Several studies have been published comparing the differential expression methods and concluded that there are substantial differences in terms of sensitivity and specificity among the methods [33-35]. We have chosen edgeR software [4] from R statistical package for gene expression analysis. In our source code for MAP-RSeq pipeline, we have Perl, R scripts and instructions that can be used post MAP-RSeq run for differential expression analysis.

### Expressed SNVs (eSNVs) from RNA-Seq

**Figure 5:**
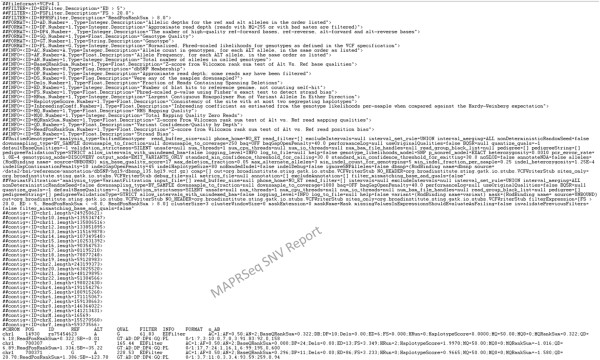
**Screenshot of a MAP-RSeq VCF files after VQSR annotation.** An example of SNV data representation from MAP-RSeq runs.

**Figure 6:**
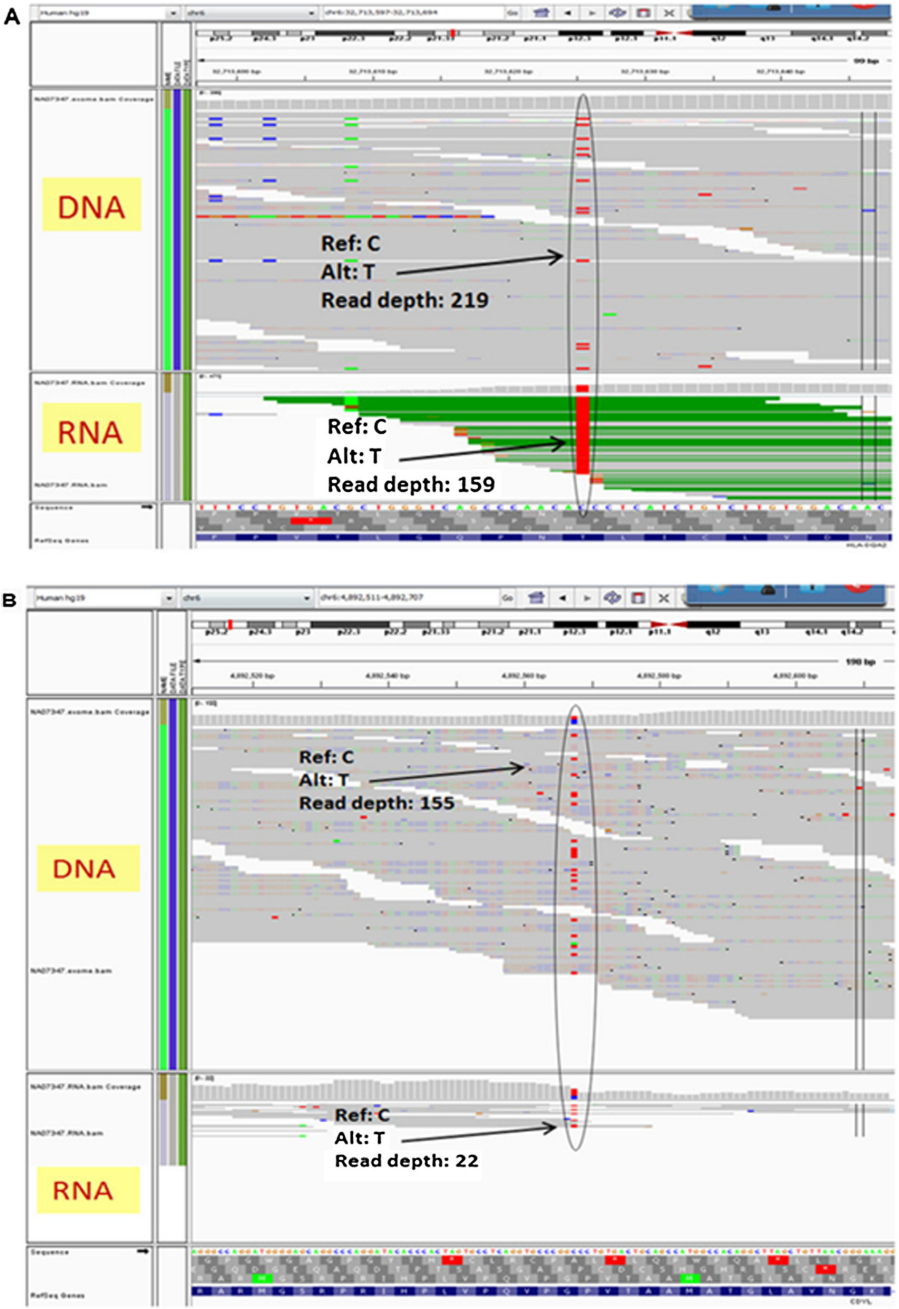
**Examples of SNVs called in RNA and DNA data for NA07347.** An IGV screenshot representation of SNV regions for the 1000 genome sample NA07347 **A)** at high read depths called in RNA when compared to exome/DNA data and **B)** at low read depth called in RNA when compared to exome/DNA data.

After filtering out multiple mapped and fusion reads, the MAP-RSeq calls SNVs using UnifiedGenotyper v.1.6.7 and VariantRecalibrator from Genome Analysis ToolKit (GATK) with the alignment files generated by Tophat. The UnifiedGenotyper from GATK is a single nucleotide variant (SNV) and indel caller developed by the BROAD institute [18]. SNVs are further annotated by the variant quality score recalibration (VQSR) method. The annotated SNVs are further filtered based on read quality (QD), coverage (DP), strand bias (FS), and positional bias (ReadPosRankSum) to identify true variants.A 1000 genome sample (NA07347) with both exome and RNA-Seq data was used to validate the SNV calling module of MAP-RSeq workflow. A concordance rate of 95.6% was observed between the MAP-RSeq SNV calls and the exome sequencing variant calls for NA07347. Figure  5 shows a screenshot of the MAP-RSeq variant calling file. Confident variant calls from MAP-RSeq workflow at high and low read depths of sequencing are shown in Figure  6A and 6B respectively.

### Fusion transcript detection

**Figure 7:**
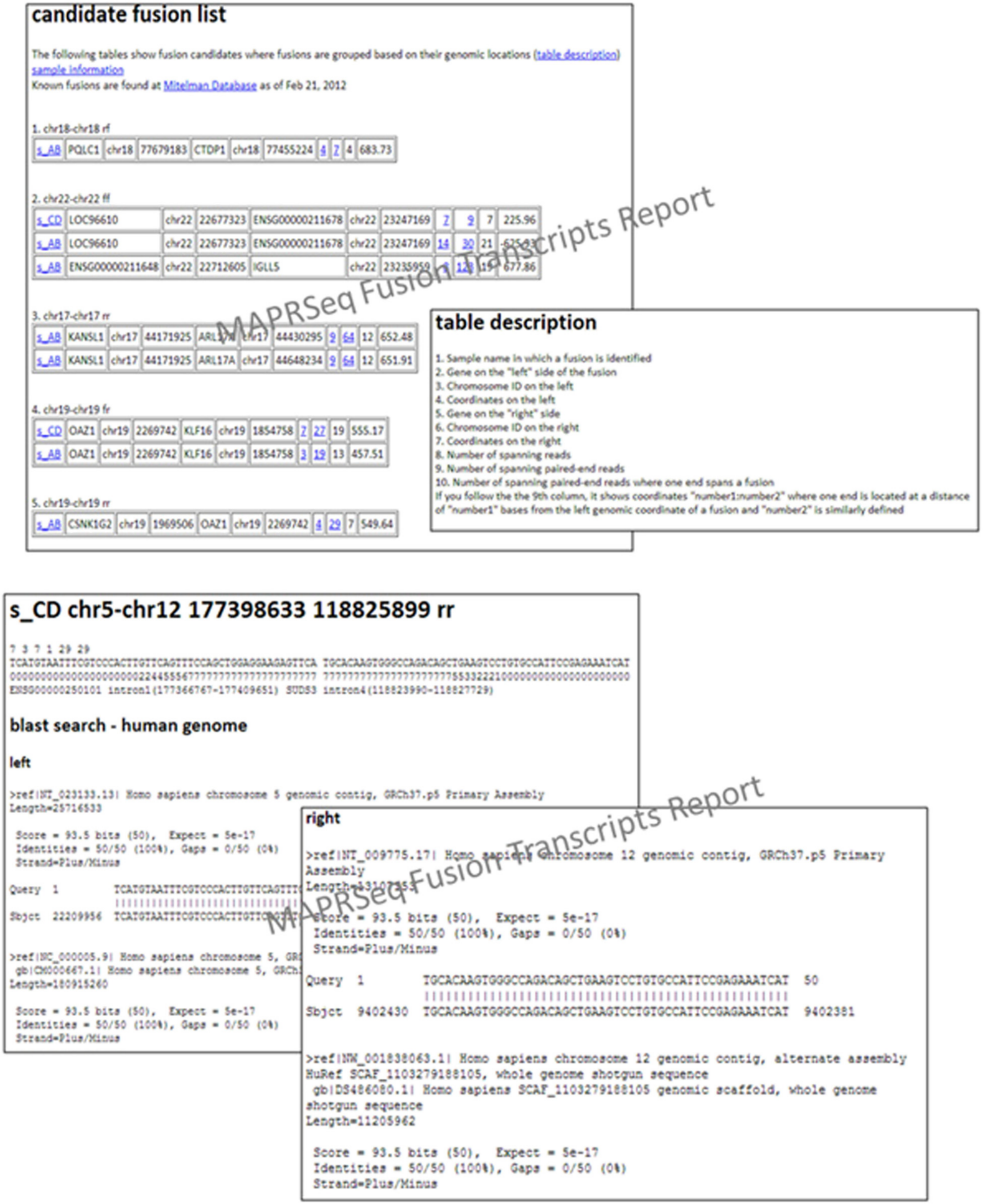
**Fusion transcripts reported by MAP-RSeq.** An example of the fusion transcripts output file from MAP-RSeq workflow.

The TopHat-Fusion algorithm identifies fusion transcripts accurately [36]. MAP-RSeq uses the TopHat-Fusion algorithm and provides a list of expressed fusion transcripts. In addition to the output from TopHat-Fusion, we have implemented modules to visualize fusion transcripts using circos plots [16]. Fusion transcript candidates are reported and summarized by MAP-RSeq. As shown in Figure  7, intra and inter fusion transcripts along with annotations are provided for each sample by the workflow. A circos plot is generated to visualize fusion transcripts across an entire RNA-Seq run (see Additional file 1). MAP-RSeq also generates 5′–3′ fusion spanning sequence for PCR validation of fusion transcripts identified. These primer sequences can be selected by researchers to validate the fusion transcripts.

### Summarization of data and final report

The workflow generates two main reports for end users: 1) summary report for all samples in a run with links to detailed reports and six QC visualizations per sample 2) final data report folder consists of exon, gene, fusion and expressed SNV files with annotations for further statistical and bioinformatics analysis.

A screenshot of an example report from MAP-RSeq is shown in Figure  2. A complete form of the report is presented in the additional file provided (see Additional file 1). Detailed descriptions of the samples processed by MAP-RSeq along with the study design and experiment details are reported by the workflow. Results are summarized for each sample in the report. Detailed quality control information, links to gene expression counts, exon counts, variant files, fusion transcript information and various visualization plots are also reported.

## Conclusions

MAP-RSeq is a comprehensive simple-to-use application. MAP-RSeq reports alignment statistics, in-depth quality control statistics, gene counts, exon counts, fusion transcripts, and SNVs per sample. The output from the workflow can be plugged into other software or packages for subsequent downstream bioinformatics analysis. Several research and clinical projects at the Mayo Clinic have used the gene expression, SNVs and fusion transcripts reports from the MAP-RSeq workflow for a wide range of cancers and other disease-related studies. In future, we plan to extend our workflow such that alternate splicing transcripts and non-coding RNAs can also be obtained.

## Availability and requirements

**Project name**: MAP-RSeq

**Project home page**: http://bioinformaticstools.mayo.edu/research/maprseq/

**Operating system(s)**: Linux or VM

**Programming language**: PERL, Python, JAVA, R and BASH

**Other requirements**: none

**License**: Open Source

**Any restrictions to use by non-academics**: none

## Competing interests

The authors declare they have no competing interests.

## Authors’ contributions

KRK , JPK, AET, YA conceived of the project, KRK, AAN, JB, JID, DO, MB, XT, SB, SM, HS, AET, YA, and JPK designed the project, KRK, AAN, JB, JID, DO, MB, JN, XT, SB, JD, SM evaluated software capabilities, KRK, AAN, JB, JID, DO, MB, JN, XT, SB, JID, SM and provided feedback on website implementation. KRK, AAN, JB, JID, DO, MB, JN, XT, SB, JID implemented the project. KRK, AAN, JB, DO, MB, wrote the manuscript. All authors read and approved the final manuscript.

## Supplementary Material

Additional file 1 **Summary report from the MAP-RSeq workflow.** Complete report in HTML format which summarizes the study design, alignment and expression statistics per sample, links to pre- and post-QC plots as well as to the resulting files on gene and exon expression, fusion transcripts and SNVs identified per sample. Click here for file
