# Supplementary material for: MAP-RSeq: Mayo Analysis Pipeline for RNA sequencing
Source: BMC Bioinformatics. 2014 Jun 27;15:224. doi: 10.1186/1471-2105-15-224 (PMC4228501; doi:10.1186/1471-2105-15-224)
Supplement: Additional file 1 — Summary report from the MAP-RSeq workflow. Complete report in HTML format which summarizes the study design, alignment and expression statistics per sample, links to pre- and post-QC plots as well as to the resulting files on gene and exon expression, fusion transcripts and SNVs identified per sample. [file 1471-2105-15-224-S1.pdf]

Summary report from the MAP-RSeq workflow . Report in .html format which summarizes the study design, alignment and expression statistics per sample, links to pre- and post-QC plots as well as to the resulting files on gene and exon expression, fusion transcripts and SNVs identified per sample.

ßßß Mayo BIC PI Support ßßß

Contents

- 1 Project Title
- 2 Initial Meetings and Timeline
- 3 Project Description
  - 3.1 Goals of the project
- 4 Analysis plan
- 5 Received Data
  - 5.1 Sample Summary
- 6 Results Summary
  - 6.1 QC steps
  - 6.2 Statistics based on per sample analysis
- 7 Appendix

I. Project Title :

NGS Bioinformatics for mrnaseq sequencing

II. Project Description

1. Background

[TOP]

ITEM	DESCRIPTION
Disease Type	Cancer
Number of Samples	2
Read Length	50
PairedEnd(PE)/SingleRead(SR)	PE
Genome Build (hg18/hg19)	hg19
StartDate	06/12/2013
EndDate	06/14/2013

2. Study design

- **What are the samples?**
  This section includes all information available from the investigator about the samples
- **Goals of the project**
  This section includes specific goals set by the investigator for the project

III. Analysis Plan

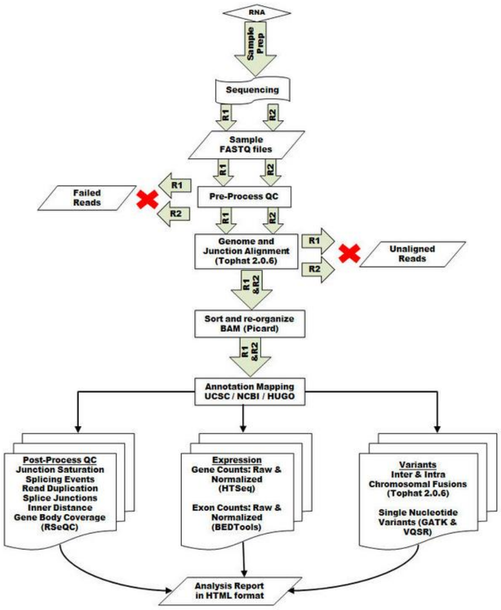

[TOP]

IV. Received Data

- **Run Name**
  130605_SN7001122_0123_BH0BEKADXX
- **Sample Summary**

Lane	Index	Sample Names
1	-	s_AB
2	-	s_CD

V. Results Summary:

- **QC steps - FastQC-report**
  FastQC aims to provide a simple way to do some quality control checks on raw sequence data coming from high throughput sequencing pipelines. It provides a modular set of analyses which you can use to give a quick impression of whether your data has any problems of which you should be aware before doing any further analysis.
  FastQC Reports
- **Statistics based on per Sample Analysis (ColumnDescription)**
  Analysis is carried out using fastq sequence files as input and generates output tables. For paired-end runs, the tables contain counts for each sample combined from both reads.

SAMPLE(S)	TOTAL READS	USED READS	MAPPED READS	MAPPED READS (GENOME)	MAPPED READS (JUNCTION)	GENE COUNT	EXON COUNT	SNVS IDENTIFIED
s_AB	294,030,280	282,256,623	262,321,294 (89.2)	236,598,852 (80.5)	25,722,442 (8.7)	163,745,488 (55.7)	185,350,787	292,827
s_CD	367,467,944	366,429,975	350,734,057 (95.4)	316,656,109 (86.2)	34,077,948 (9.3)	195,569,950 (53.2)	236,985,171	383,190

VI. Results Delivered

The following three sets of tables are delivered and column description is available in Appendix.

- **Exon table: contains counts for the number of times an exon has been detected**
  count (raw) = sum of exon read counts

  count (RPKM)

[TOP]

- **Gene table: contains counts for the number of times a gene copy has been detected**
  count (raw) = sum of exon read counts, with an exception that if reads start in different exons of the same gene twice, these are counted only once for the gene
- **SNV reports: contains Single Nucleotide Variants (SNV) called using GATK software**
  sample.gatk.vcf = raw SNV calls for each sample

  sample.filter.vcf = SNV calls annotated using VQSR filters
- **RSeQC Plots:**

Sample Name	Junction Saturation	Splicing Junction	Splicing Event	Inner distance	Read duplication	Gene body coverage
S_AB	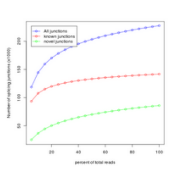	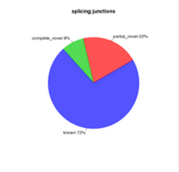	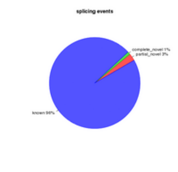	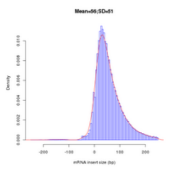	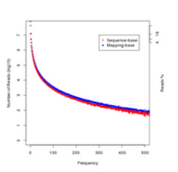	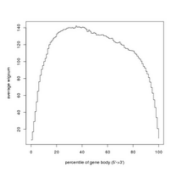
S_CD	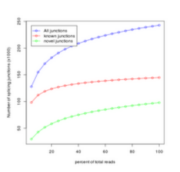	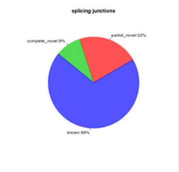	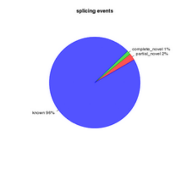	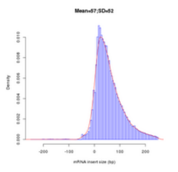	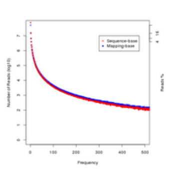	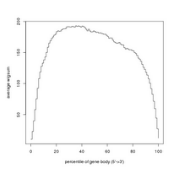

- **Tophat fusion (circos plot):**
  Tab delimited fusion results are available here

  Detail fusion report in HTML format is available here

Circos plot

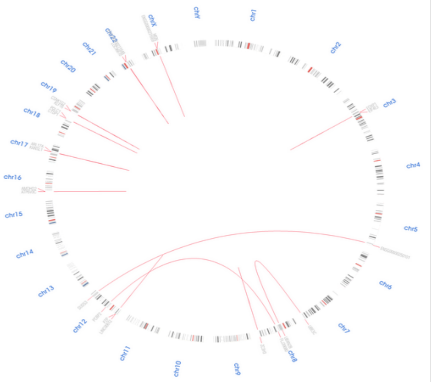

[TOP]

- **Statistics based on per Sample Analysis are recorded in the tab delimited file Details**
- **IGV Visualization**
  The SNV and INDEL annotation reports (both standard and filtered) include visualization links to IGV to enable a realistic view of the variants. Please follow steps in the following link to setup IGV (takes less than 5 minutes) and utilize this feature. IGV setup for variant visualization

VII. Useful Links

- Tophat 2
- Tophat Fusion
- HtSeq
- RSeQC
- GATK
- UCSC Genome Browser

VIII. Appendix

Full Length cDNA Sequencing (mRNA- Seq) results delivery format(Appendix)

Authorship Consideration

Advancing scientific research is a primary motivation of all bioinformaticians and acknowledgment of contribution through authorship on manuscripts arising from this analysis is one way our work is assessed and attributed. We request to be considered for authorship on any manuscripts using the analysis results provided if you believe we have made substantive intellectual contributions to the study.
